# Longitudinal analysis of developmental changes in electroencephalography patterns and sleep-wake states of the neonatal mouse

**DOI:** 10.1371/journal.pone.0207031

**Published:** 2018-11-06

**Authors:** Nicholas Rensing, Brianna Moy, Joseph L. Friedman, Rafael Galindo, Michael Wong

**Affiliations:** Department of Neurology and the Hope Center for Neurological Disorders, Washington University School of Medicine, St. Louis, Missouri, United States of America; University of Catanzaro, ITALY

## Abstract

The neonatal brain undergoes rapid maturational changes that facilitate the normal development of the nervous system and also affect the pathological response to brain injury. Electroencephalography (EEG) and analysis of sleep-wake vigilance states provide important insights into the function of the normal and diseased immature brain. While developmental changes in EEG and vigilance states are well-described in people, less is known about the normal maturational properties of rodent EEG, including the emergence and evolution of sleep-awake vigilance states. In particular, a number of developmental EEG studies have been performed in rats, but there is limited comparable research in neonatal mice, especially as it pertains to longitudinal EEG studies performed within the same mouse. In this study, we have attempted to provide a relatively comprehensive assessment of developmental changes in EEG background activity and vigilance states in wild-type mice from postnatal days 9–21. A novel EEG and EMG method allowed serial recording from the same mouse pups. EEG continuity and power and vigilance states were analyzed by quantitative assessment and fast Fourier transforms. During this developmental period, we demonstrate the timing of maturational changes in EEG background continuity, frequencies, and power and the emergence of identifiable wake, NREM, and REM sleep states. These results should serve as important control data for physiological studies of mouse models of normal brain development and neurological disease.

## Introduction

The neonatal brain experiences rapid changes that facilitate the normal development, plasticity and growth of the nervous system and also affect the pathological response to brain injury. Electroencephalography (EEG) is a powerful tool for assessing function in the normal and diseased brain [1, 2]. In contrast to the relatively stable EEG of the normal juvenile and adult brain, the neonatal and infantile EEG undergoes dramatic changes over relatively short time periods secondary to early developmental processes in brain physiology and connectivity [1–3]. These age-dependent alterations in early postnatal EEG offer a window into the underlying mechanisms that govern brain maturation. Therefore, the study and development of techniques that allow for the systematic longitudinal and serial evaluation of early postnatal EEG offer the ability to better understand immature cerebral function in healthy and disease conditions.

Animal models are critical for understanding processes underlying normal human brain development and investigating pathophysiological mechanisms of a variety of neurological disorders affecting the neonatal and infant population. While developmental changes in human EEG have been described in detail [2–7], less is known about the normal maturational properties of rodent EEG, including the emergence and evolution of sleep-awake vigilance states. For example, while a number of comprehensive developmental EEG studies have been performed in neonatal rats [8–14], due to technical limitations (e.g., smaller head size) and other factors, few developmental EEG studies in normal neonatal mice have been completed [15–17], and are more limited in their scope and focus. In particular, to our knowledge, there have been no longitudinal studies that systematically and serially evaluate the age-dependent changes in postnatal EEG in normal mice. As mice represent a common species utilized for translational research of genetic and non-genetic conditions, a comprehensive assessment of EEG characteristics and vigilance state across neonatal development utilizing a serial-single mouse recording technique would be of significant value to studies of normal brain maturation and neurological disease during important developmental time points. In this study, we have performed serial video, EEG, and EMG recordings of mouse pups from postnatal day 9 to 21 to provide a relatively comprehensive longitudinal characterization of EEG properties and vigilance state changes during this critical period of brain maturation.

## Materials and methods

### Animals

Care and use of all mice were conducted according to an animal protocol approved by the Washington University School of Medicine (WUSM) Animal Studies Committee, and consistent with National Institutes of Health (NIH) guidelines on the Care and Use of Laboratory Animals. In addition, NIH guidelines on Rigor and Reproducibility in Preclinical Research were followed, including use of randomization, blinding, both sexes, and statistical/power analyses.

Control male and female mice with a mixed genetic background (SV129/CDA/C57) were obtained from an existing colony maintained at WUSM. Although genetic background might influence EEG and sleep phenotype, the mixed background may be appropriate for future studies of genetic mouse models that involve the crossing of different parental strains. Multigravida pregnant females were acclimated to the laboratory environment 2–3 days prior to giving birth to reduce maternal stress. Date of birth was considered postnatal day 0 (P0) and litters were culled to 6–8 pups at P5.

Mice were euthanized by rapid decapitation under isoflurane anesthesia, consistent with the guidelines of the Panel on Euthanasia of the American Veterinary Medical Association.

### Electroencephalography (EEG) electrode surgery

**Fig 1:**
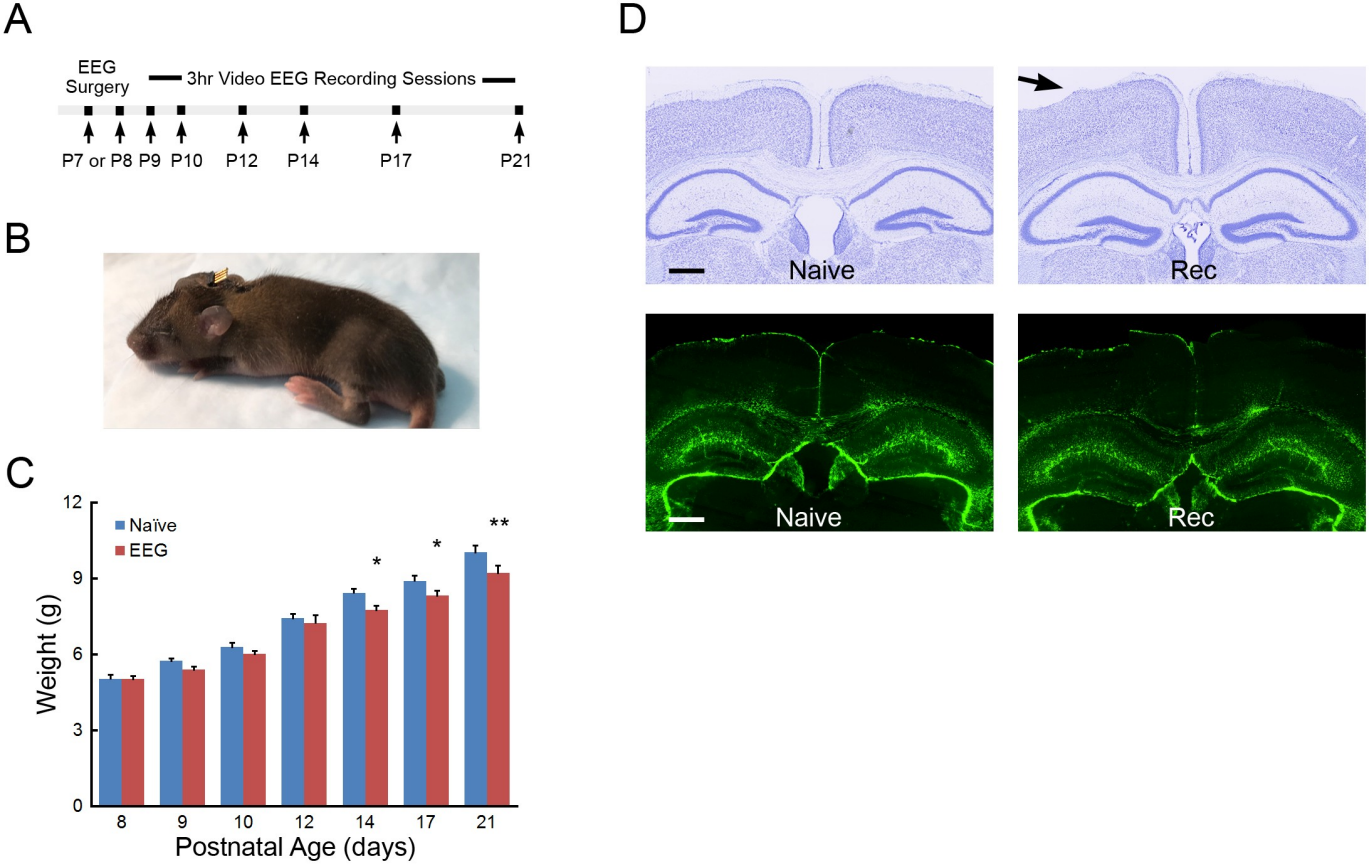
Longitudinal EEG and EMG recordings in the neonatal mouse. (**A**) Protocol summarizing the timing of the longitudinal recording sessions in individual mice. (**B**) After electrode placement surgery at P7 or P8, the attached electrode apparatus is of minimal size and the exposed pin header allows reversible connections for recording EEG and EMG while permitting pup feeding and group nesting. (**C**) Weight gain in operated and naïve mice. *p<0.05, P14 and P17, **p<0.01, P21 naïve compared to EEG recorded mice by two-way repeated measures ANOVA with Holm-Sidak posttest; n = 9–11 per age group. **(D)** Histological assessment of electrode placement and injury during EEG recording of the neonatal mouse. Top panels: Cresyl violet stained coronal sections display limited injury to underlying layer I/II cortex of naive or EEG recorded (Rec) mice caused by surgery or recoding paradigm. Bottom panels: GFAP analysis shows no signs of gliosis in the cortex or hippocampus of EEG recorded (Rec) neonatal mice compared to naïve (Naïve) controls; n = 8 Rec, n = 6 Naïve. Scale bar is 500μm.

Mouse pups received surgery for placement of EEG electrodes on P7-P8 (Fig 1A). Custom wire electrode sets were constructed using four Teflon coated stainless steel wires (76 μm bare diameter) soldered to a pin header with 1mm pitch spacing. The soldered contacts were covered with dental cement and 1mm of Teflon coating removed from the end of the exposed wires. Mice were placed under 4% isoflurane administered through a custom nose piece on a heating pad set to 36.5°C until pedal withdraw reflex ceased. The skin was prepared with betadine and alcohol wipes with isoflurane maintained at 2–2.5% for the remainder of the procedure. After a midline vertical incision to expose the skull, forceps and 3% hydrogen peroxide were used to remove any connective tissue and dry the skull for electrode placement. A hole for the frontal reference electrode was placed in the skull (anterior +0.8mm, lateral 0.5mm; bregma) using the tip of a 29g needle. The uncoated tip of the stainless steel wire was positioned approximately 1mm anterior into the burr hole in contact with the surface of the cortex. The coated portion of the wire was secured to the skull using Locktite 454 followed by small amount of dental cement (SNAP, Parkell). The two bilateral “active” recording electrodes were individually inserted and secured in place over the parietal cortex (posterior -2.3mm, lateral +/- 2.0; bregma) using the same techniques as the reference electrode. The final electrode wire was inserted in the neck muscle for nuchal electromyography (EMG) recordings with the coated portion of the wire bent to follow the contour of the head. The exposed skull and any visible wires were covered in a layer of dental cement and the pin header angled and secured to the head to allow the pup to nurse. The skin was sutured around the exposed pin headers and tissue glue (Vetbond, 3M) used to close the remainder of the incision. A small amount of dental cement was used to cover and shape the electrode array to the contour of the head (Fig 1B). The weight of the electrode assembly was approximately 50 mg, with an additional 50–100 mg of dental cement added to secure the electrodes to the skull. Neonatal pups received Buprenorphine (0.1mg/kg) and recovered in a warmed chamber for one hour prior to returning to the dam. In addition to the compact size of the electrode assembly, the one-hour recovery period increased re-acceptance of the pup by the dam. Furthermore, maternal care to the electrode-implanted pups was optimized by preconditioning the dam with removal and reintroduction of a sham-operated pup.

### Video-EEG monitoring

Mouse pups were separated from the dam and received serial three-hour video-EEG monitoring sessions between P9 and P21 (daily from P9-P14, P17 and P21) during the light phase of a 12 hr light/12 hr dark cycle. Mouse pups were placed in heated Plexiglas recording chamber with the temperature adjusted daily in order to maintain body temperature near 36°C and compensate for greater thermoregulation as the pups aged. A custom flexible cable was attached to the exposed pin header on the freely moving pup and connected to single channel AC amplifiers (P511, Grass). Bilateral cortical EEG signals were acquired using a referential montage and the signals amplified at 20,000X with highpass (1Hz) and lowpass (100Hz) filters applied. EMG signals were filtered with highpass (10Hz) and lowpass (300Hz) filters. EEG and EMG signals were digitized at 400Hz (MP150; Biopac) and pups were recorded in up to 3-hour sessions. After the recording session, pups were immediately weighed and returned to the dam.

### EEG review and vigilance state scoring

Bilateral EEG and nuchal muscle EMG files (.edf) were imported into AdInstruments LabChart software and a digital bandpass (1-35Hz EEG; 10-100Hz EMG) filter applied for review. Neonatal mice P9-P14 were manually scored in 5 second epochs and vigilance state scored as awake, non-REM sleep (NREM) or REM sleep (REM) using a combination of the EEG, EMG and respective spectral power representations. As the mice aged and produced longer sleep cycles, older P17 and P21 mice were scored in 10 second epochs. Vigilance state scoring parameters were assessed and defined in the older mice based on standard criteria for adult rodents [12–14, 18], and similarly applied to the younger neonatal mice when such states were recognizable. Wakefulness was defined as periods of cyclic lower amplitude mixed frequency EEG and high tone muscle activity EMG for greater than half of the epoch duration. Brief arousal periods around 3-5sec with high muscle tone during sleep transitions were also scored as awake. EEG periods dominated by higher amplitude delta wave activity with nuchal muscle atonia were scored as NREM sleep epochs. In the younger neonatal mice, NREM sleep periods occasionally contained brief movement (<1 sec.) but the EEG contained higher amplitude delta and were subsequently labeled as NREM. REM sleep consisted of periods of semi-uniform theta activity or mixed frequency EEG with muscle atonia and/or muscle atonia with brief myoclonic twitches. In the younger neonatal mice prior to development of theta rhythms, the above REM criterion was used along with the absence of slow wave delta activity. EMG and video data were used to help exclude artifacts.

### Sleep analysis

Percentage of time spent in each vigilance state was tabulated using LabChart as a function of recording time. Vigilance state transitions were calculated using the scored comment and averaged per hour. Scored transitions were used to determine the bout durations by calculating the state specific time spent between vigilance state changes. The percentage of total power in delta or theta ranges were calculated using sorted vigilance state spectral data in the 1–4 Hz and 4–8.5Hz frequency ranges, respectively, and excluded epochs containing obvious artifact.

Young neonatal mice have varying periods of low voltage discontinuous EEG. As the mice mature the frequency of the discontinuous periods decreases and the duration becomes shorter. Qualitative assessments of discontinuous EEG were manually selected using standardized one-hour raw EEG traces from P9 to P17 pups. The duration of discontinuous periods was selected from non-artifact EEG with a greater than 50% reduction in amplitude from baseline and with an average voltage below +/- 10 μV and deviation of +/- 20 μV. A return in amplitude to baseline levels indicated the end of the discontinuous period.

### Total power FFT

Total power fast Fourier transforms (FFT) were calculated using LabChart software from 1-20Hz with 512 bin size and a Hann (cosine-bell) data window using spectral data extracted from each epoch. Epochs with obvious movement artifact or epochs with vigilance state and age specific artifact thresholds were excluded. FFTs were sorted by vigilance state and left and right EEG power data were averaged and displayed with a 1Hz frequency resolution.

### Histology

Operated and naïve mouse pups aged P22-P25 were perfusion-fixed with 4% paraformaldehyde and cut into 45 μm sections with a freezing microtome. Sections near the placement of electrodes were stained with 0.5% cresyl violet acetate to assess electrode location and gross morphological condition of the underlying cortex or damage caused by the implanted electrodes or other methodological processes. Immunohistochemistry was performed for GFAP (1:500; #3670, Cell Signaling Technology) followed by labeling with secondary antibody Alexa-488 conjugated goat anti-rabbit IgG (#A11034, Life Technologies) and DAPI nuclear stain to assess gliosis near the resting electrodes or along the electrode track. In addition, sequential sections were stained with Fluro-Jade C (FJC) and DAPI to assess cellular necrosis or cellular damage near the electrode site.

Images were acquired with a Hamamatsu NanoZoomer 2.0 pathological microscope and GFAP-immunoreactive and FJC cells in neocortex and hippocampus were counted by a blinded investigator. In images from coronal sections at ~ 2 mm posterior to bregma and ~ 500μm from midline, regions of interest were marked in neocortex by a 200 μm wide box spanning from the neocortical surface to the bottom of layer VI. Hippocampal gliosis and necrosis were quantified using 1mm x 500μm area between the CA1 pyramidal cell layer and along the granular layer of the dentate gyrus. GFAP-immunoreactive cells and FJC positive cells were counted bilaterally in the regions of interest from one section per mouse from a total of 6–8 mice per group in a blinded fashion.

### Statistics

All statistical analysis was performed using SigmaStat 3.5 software. Quantitative differences in weight between naïve and operated mice were analyzed by two-way repeated measures ANOVA with multiple comparisons using the Holm-Sidak method. Student’s t-test was used to compare histological measures between naïve and operated mice. Quantitative differences between ages were analyzed by one-way repeated measures ANOVA with Tukey post hoc tests. Quantitative differences in FFT generated frequency bins of normal distributed data were analyzed by one-way ANOVA with Bonferroni post hoc tests and non-parametric frequency data analyzed by Kruskal-Wallis One Way Analysis of Variance on Ranks with Dunn’s post hoc tests. 1 Hz bins in the frequency range of 1–20 Hz were analyzed; multiple comparison corrections were not made because multiple, consecutive bins were found to be significant, which exceeds the predicted chance of random false positives. Quantitative data are expressed as mean ± SEM. Statistical significance was defined as p < 0.05.

## Results

### A novel method for longitudinal EEG and EMG analysis of neonatal mice

We developed a method to allow longitudinal assessment of EEG background and wake-sleep states by serial EEG and EMG recordings from neonatal mice between P9 to P21. Following surgical electrode implantation at P7 or P8, the exposed pin header secured on the pups’ head allowed for reversible connection of the pup in daily three-hour recording sessions from P9-P14, P17, and P21, with return to the dam in between sessions (Fig 1A and 1B). Actual duration of recorded data varied due to occasional disconnection of the recording cables from the head as the pups aged (average recording duration; P9 2.7 +/- 0.4 hours, P21 2.1 +/- 0.4 hours). No immediate adverse effects were qualitatively observed following electrode placement, with the pup resuming nesting and normal feeding behavior. With occasional exceptions, the dam demonstrated no adverse behavior towards pups with implanted electrodes. The body weight of-recorded pups showed lower weight gain between P14-P21, compared with naïve littermates, which likely related to being separated for 3 hours a day with decreased time for feeding and increased exposure isolated from the litter, particularly during light phase recording [19, 20]. However, EEG recorded pups had continued growth during the P14-P21 periods and weights were within <10% difference of naïve littermates and within the weight range of several strains at weaning [21] (Fig 1C). No obvious pathophysiological changes in EEG were observed, such as electrographic seizures or focal slowing.

Histological analysis confirmed the expected EEG electrode placement and revealed that the wire electrodes sometimes caused mild to moderate compression and superficial tearing of layer I and/or layer II neocortex in areas near the resting electrodes (Fig 1D top). However, this limited damage was not accompanied by activated gliosis assayed by GFAP immunoreactive quantification of the neocortex (24.8 ± 3.0 GFAP-positive cells/ROI in operated mice vs. 26.3 ± 4.6 in naïve; p = 0.70) or hippocampal areas (40.3 ± 6.4 GFAP-positive cells/ROI in operated mice vs. 46.4 ± 6.5 in naïve; p = 0.38; n = 6–8; Fig 1D bottom). Furthermore, in assaying neuronal death by FJC, no FJC positive cells were detected in most sections in both operated and naïve mice and there was no significant difference in the low number of FJC-positive cells between the two groups (neocortex: 0.9 ± 0.4 FJC-positive cells/ROI in operated mice vs. 0.3 ± 0.4 in naïve, p = 0.14; hippocampus: 0.4 ± 0.2 FJC-positive cells/ROI in operated mice vs. 0.08 ± 0.1 in naïve, p = 0.14; n = 6–8 per group).

### Identification of vigilance state and EEG characterization in the developing neonatal mouse

**Fig 2:**
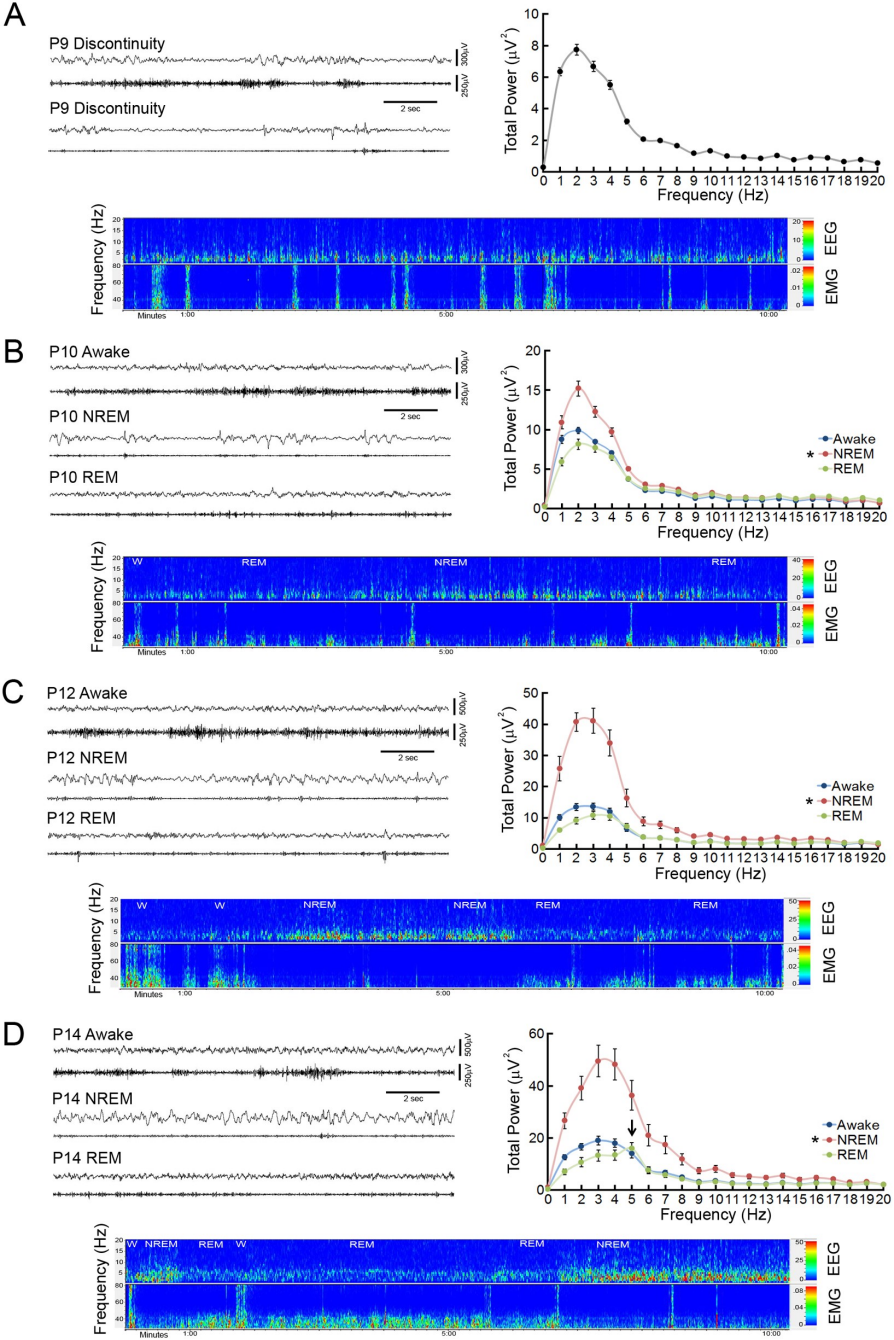
Identification of vigilance states in the neonatal mouse. (**A**) Representative 15 second EEG and nuchal EMG traces from a postnatal day 9 (P9) mouse exhibit a discontinuous EEG pattern during periods of high muscle tone (upper traces) and muscle atonia (lower traces). A ten minute EEG/EMG CDSA displays bursts of EEG activity separated by brief periods of suppressed EEG power and limited slow wave activity. Despite intermittent bursts of EMG activity, EEG shows no definite evidence of qualitative state changes. The P9 FFT displays the mean total power within the frequency for the entire recording period. (**B**) Representative P10 EEG and EMG traces display initial evidence of state change, with low amplitude, relatively continuous EEG activity and prominent EMG activity during the awake state, higher amplitude discontinuous bursts of prominent slow wave activity on EEG and decreased EMG activity during NREM, and low amplitude continuous EEG activity with suppressed EMG with intermittent myoclonic activity during REM sleep. The CDSA displays state differences in short, defined vigilance patterns with multiple sleep/wake cycles (W- Awake; NREM- NREM sleep; REM- REM sleep; not all vigilance epochs labeled) of a P10 neonatal mouse. The P10 power FFT displays a significant increase in power (1-7Hz) during NREM labeled epochs. *p<0.05, compared to awake/REM by ANOVA with Tukey; n = 8. (**C**) Representative P12 EEG, EMG, and CDSA traces display well-defined distinction between different vigilance state patterns, with limited discontinuity during NREM sleep. The P12 power FFT displays a two-fold increase in delta power (1-4Hz) with an increase in overall power (1-17Hz) during NREM sleep episodes. *p<0.05, compared to awake/REM by Kruskal-Wallis with Dunn’s; n = 9. (**D**) Representative P14 EEG, EMG and CDSA traces display clear vigilance state patterns. The P14 power FFT displays significant increase in delta power during NREM sleep and the development of a 5Hz peak during REM sleep epochs (arrow). *p <0.05, versus awake/REM by one-way ANOVA; n = 8.

Video, EEG and EMG data, along with corresponding power color density spectral array (CDSA) patterns, were used to determine vigilance states and EEG characteristics in the developing neonatal mouse from P9 to P21. Daily recordings were performed between P10-P14, but did not generally demonstrate statistically significant differences in analysis between consecutive days, so for simplicity data for P11 and P13 are not shown in the subsequent results and figures. In P9 mice, cortical EEG exhibits primarily a discontinuous pattern, characterized by brief segments of higher amplitude, lower frequency activity separated by periods of low amplitude, relatively suppressed activity (Fig 2A). As the discontinuous pattern predominates during both periods of nuchal muscle tone with or without brief myoclonic jerks and periods of muscle atonia, there is no definite evidence of state change on EEG at P9. A power CDSA representation also confirms a lack of vigilance state change, demonstrating invariant, brief epochs of EEG activity during times of both EMG bursts and atonia.

By P10, initial evidence of distinctive cortical EEG and nuchal EMG vigilance state patterns develop (Fig 2B). The awake state is characterized by continuous periods of low amplitude mixed frequency EEG associated with active nuchal muscle tone. Sleep is comprised of two distinct EEG patterns during periods of muscle atonia: NREM features discontinuous, higher amplitude slow wave activity and REM is characterized by continuous periods of low amplitude mixed frequency EEG with intermittent myoclonic twitching.

In P12 mice, further maturation of cortical EEG patterns continues, making the distinction between vigilance states clearer (Fig 2C). Slow wave activity in NREM sleep periods demonstrate briefer periods of discontinuity with an overall increase in amplitude (Table 1). Sleep cycles continue to lengthen with longer periods of NREM and REM sleep segments. By P14 and beyond, continuous EEG activity is present in all vigilance states (Fig 2D). REM episodes demonstrate an increase in theta activity, which initially develops in the lower range of the theta frequency band around 5Hz.

**Table 1:** Quantitative assessment of discontinuous EEG in the developing neonatal mouse.

Age	Suppressed (Discontinuous) EEG Activity						
	# of periods/hour			duration (sec)			
P9 (n = 10)	87.9 +/- 15.9*			3.9 +/- 0.5*			
P10 (n = 8)	53.5 +/- 9.0^#^			2.2 +/- 0.1^#^			
P12 (n = 9)	5.5 +/- 1.3			1.0 +/- 0.1			
P14 (n = 8)	0.8 +/- 0.6			0.2 +/- 0.1			

*p<0.05 compared to P10, P12 and P14;

^#^p<0.05 compared to P12 and P14 by one-way repeated measures ANOVA with Tukey multiple comparisons posttest.

Overall, the EEG evolves from an invariant low amplitude discontinuous pattern into distinct vigilance state patterns with increasing continuity and power. Cortical EEG activity in P9 neonatal mice is discontinuous, with 87.9 +/- 15.9 discontinuous periods per hour and a mean 3.9 second +/- 0.5 duration (Table 1). Between P10 and P14, a progressive reduction occurs in the number of discontinuous EEG periods and duration of the discontinuity, with continuous EEG in all vigilance states at P14. Based on total power FFTs, P9 mice have relatively low overall power and exhibit no state change (Fig 2A). With P10 mice and beyond, total power FFTs were calculated for each vigilance state (Fig 2B–2D). Total power increases primarily in the 1-6Hz frequency ranges of QS/NREM sleep epochs compared to awake and AS/REM in P10 mice. At P12 NREM sleep total power increases further in the 1-17Hz frequency ranges compared to awake and REM epochs. In P14 mice, an increase in delta power continues in NREM compared to awake and REM epochs and a specific 5Hz theta peak develops during REM sleep epochs.

### Developmental changes in frequency, transitions, and bout duration of vigilance states in the neonatal mouse

**Fig 3:**
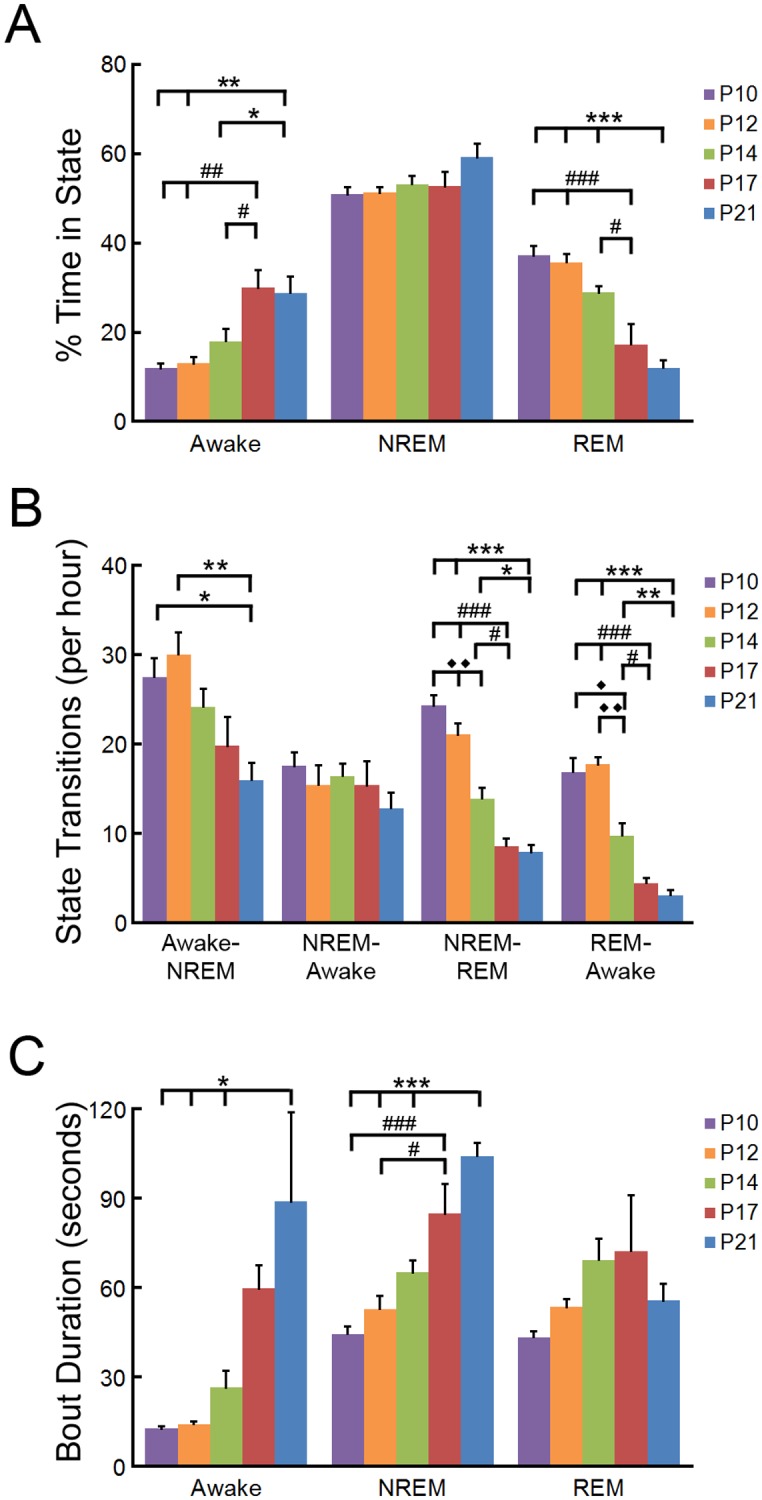
Developmental changes in the percentage of time, number of transitions, and bout durations of vigilance states of the neonatal mouse. (**A**) Percentage of recorded time spent in vigilance states of P10 through P21 mice. Older mice display a significant increase in wakefulness and decrease in REM sleep compared to P10, P12, and P14 neonates. p<0.001 compared to ***P21; ###P17, p <0.01 compared to **P21; ##P17, p<0.05 compared to *P21; #P17 by one-way repeated measures ANOVA with Tukey; n = 8–9 mice per group. (**B**) The number of vigilance state transitions of P10 through P21 mice. The number of vigilance state transitions into sleep and the number of REM transitions decrease in the developing mouse. p<0.001 compared to ***P21; ###P17, p <0.01 compared to **P21; ◆◆P17, p<0.05, compared to *P21; #P17; ◆P14 by one-way repeated measures ANOVA; n = 8–9 mice per group. (**C**) The bout duration of vigilance states of P10 through P21 mice. The bout duration of wakefulness and NREM sleep increases during development. p<0.001 compared to ***P21; ###P17, p<0.05 compared to *P21; #P17 by one-way repeated measures ANOVA with Tukey; n = 8–9 mice per group.

After vigilance state patterns develop in young neonatal mice, wakefulness comprised relatively short portion of the overall recorded time of P10 and P12 mice but increased significantly through P17-P21 (Fig 3A). By comparison, neonatal mice spend a large percentage of recorded time in sleep. Although the percentage of NREM sleep appears to show a trend toward an increase throughout neonatal development, recorded time in NREM sleep remains similar across ages. In young neonates, REM sleep constitutes a larger portion of sleep compared to older mice, as the percentage of time spent in REM decreases by P17 compared to P10, P12, and P14 pups.

Prior to P14, sleep cycles are short and neonates frequently transition between vigilance states. As the pups age, fewer vigilance state transitions occur, including transitions from wakefulness into NREM, from NREM into REM, and from REM into wakefulness (Fig 3B). There is a corresponding increase in bout durations in awake and NREM states with age, although the bout durations of REM periods are similar throughout the recording period (Fig 3C).

### Developmental changes in EEG power of vigilance states in the neonatal mouse

**Fig 4:**
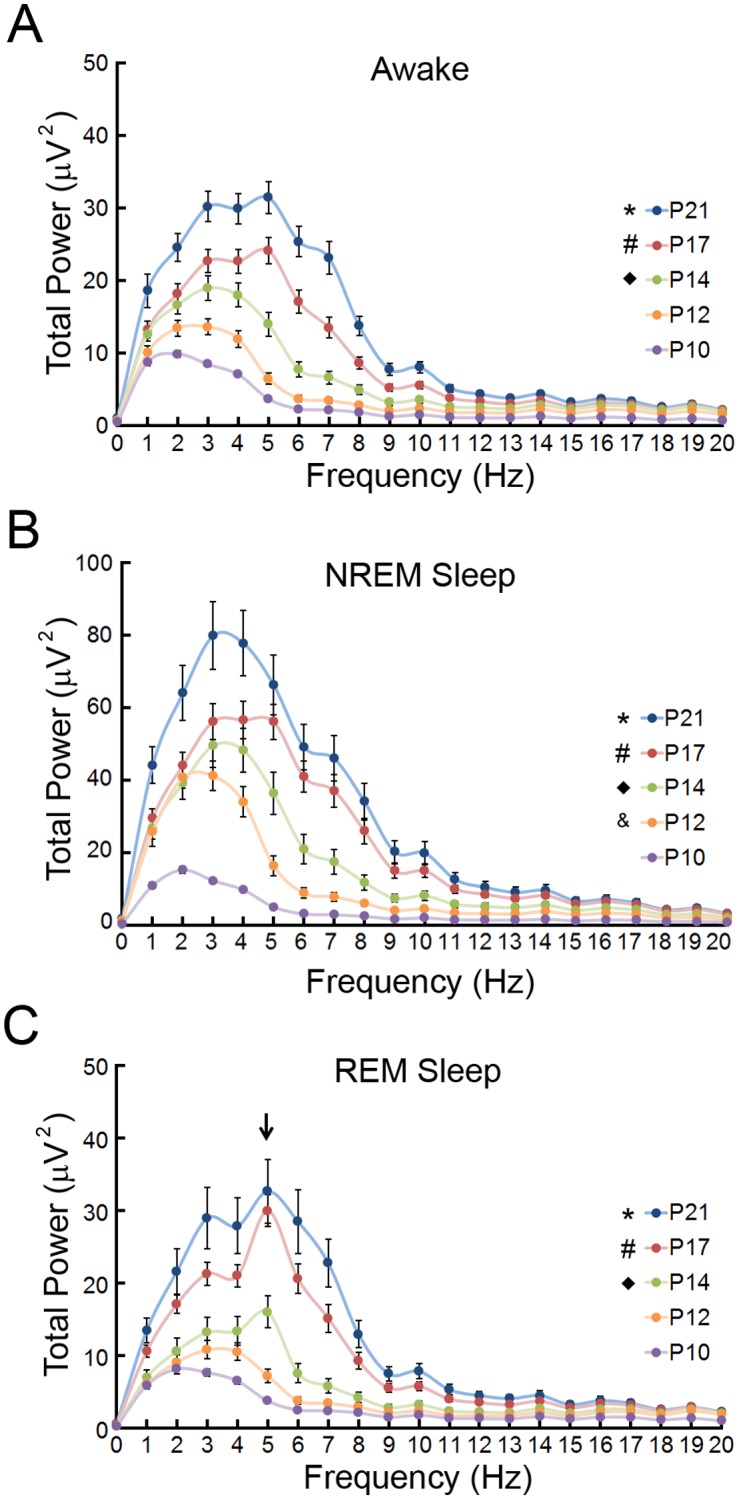
Developmental changes in total power FFT during different vigilance states of the neonatal mouse. (**A**) During wakefulness, a significant increase in total power across frequencies occurs as the mouse ages. *p<0.05, P21 compared to P10 (0–20 Hz), P12 (0–13 Hz); #p<0.05 p17 compared to P10 (2–20 Hz) P12 (5–7 Hz); ◆p<0.05 P14 compared to P10 (3–6, 15–20 Hz) by Kruskal-Wallis with Dunn’s; n = 8–10. (**B**) During NREM sleep, a significant increase in delta power occurs at P12 and power across all frequencies increases as the mouse ages. *p<0.05, P21 compared to P10 (0–20 Hz), P12 (4–14 Hz); **#**p<0.05 p17 compared to P10 (0–20 Hz) P12 (6–7 Hz); ◆p<0.05 P14 compared to P10 (0-18Hz); ***&***p<0.05 P12 compared to P10 (1–2 Hz) by Kruskal-Wallis with Dunn’s; n = 8–10 mice per group. (**C**) During REM sleep, a significant increase in theta power occurs at P14 and power across all frequencies increases as the mouse ages. *p<0.05, P21 compared to P10 (0–20 Hz); P12 (0–12 Hz); **#**p<0.05 p17 compared to P10 (0–19 Hz) P12(5–8 Hz); ◆p<0.05 P14 compared to P10 (5 Hz) by Kruskal-Wallis with Dunn’s; n = 8–10 mice per group.

FFT are useful to breakdown EEG waves into their respective amplitude/frequency components. Vigilance state patterns present after P10 provide distinct EEG power distributions. As neonatal mice mature, an overall increase in total power develops independent of vigilance state (Figs 2 and 4). In the awake state, a significant increase in power occurs across the frequency spectrum (0-20Hz), but most prominently in the 3-6Hz range, starting at P14 (Fig 4A). In NREM sleep periods, a significant increase in lower delta power (1-2Hz) occurs at P12, with an increase in power across frequencies (1-18Hz) at P14 (Fig 4B). During REM sleep episodes, similar increases in power across multiple frequencies occur with age, but there is a specific development of distinctive theta rhythm predominantly at 5–6 Hz starting at P14 (Fig 4C).

**Fig 5:**
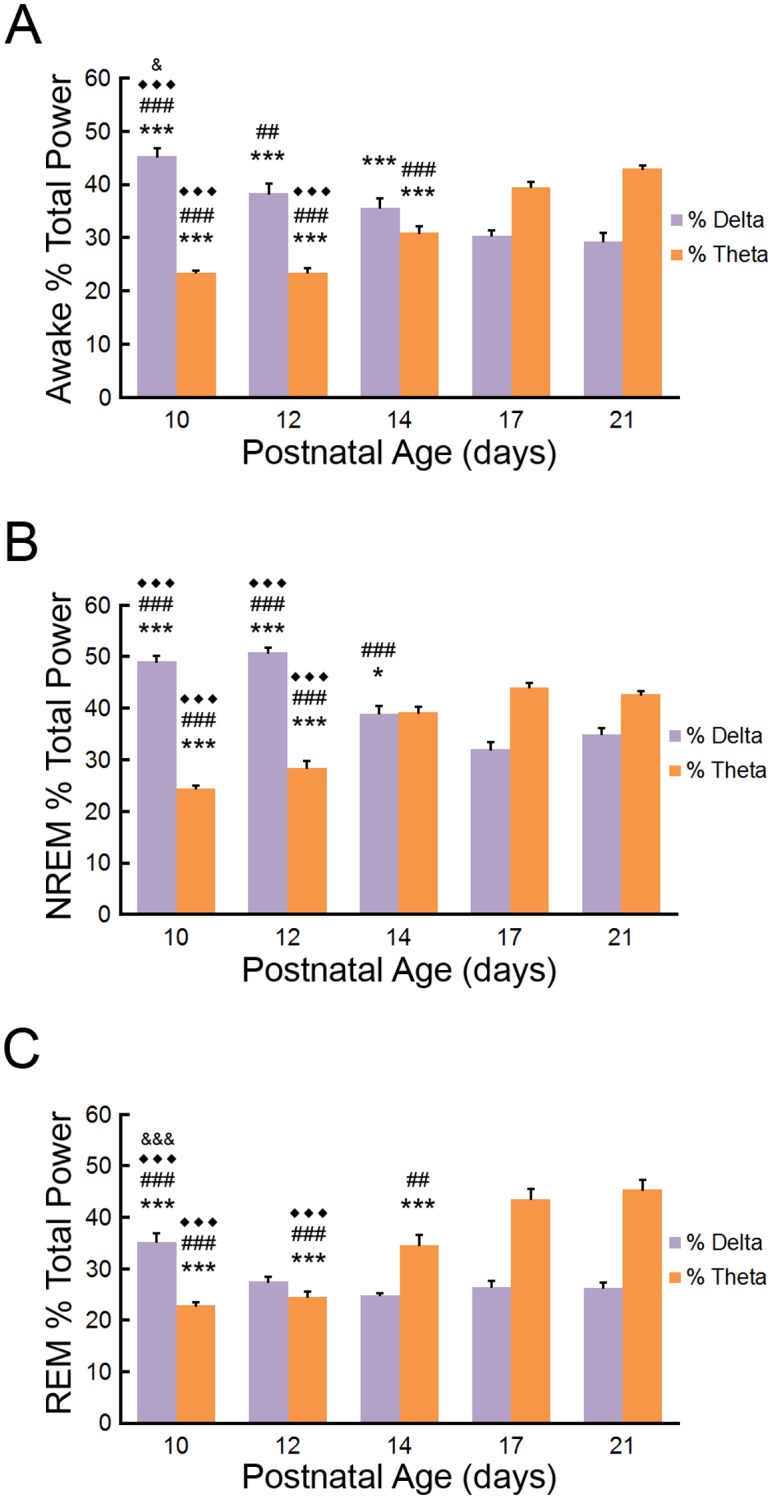
Developmental changes in percentage of EEG total power within the delta and theta frequency ranges during vigilance states of the neonatal mouse. (**A**) During wakefulness, percentage of delta (1-4Hz) in EEG decreases until P17 and percent theta (4–8.5Hz) increases starting at P14. p<0.001, compared to ***P21; ### P17; ◆◆◆ P14; p<0.01 compared to ## P17; p<0.05 compared to ^***&***^P12 by one-way repeated measures ANOVA with Tukey; n = 8–9 mice per group. (**B**) During NREM sleep, percentage of delta power decreases until P17 and percentage of theta power increases at P14. p<0.001, compared to ***P21; ### P17; ◆◆◆ P14; p<0.05 compared to *P21 by one-way repeated measures ANOVA with Tukey; n = 8–9 mice per group. (**C**) During REM sleep, percentage of delta power levels decreases after P10 and percentage of theta power increases after P12. p<0.001, compared to ***P21; ### P17; ◆◆◆ P14; ^***&&&***^ P12; p<0.01 compared to ## P17 by one-way repeated measures ANOVA with Tukey; n = 8–9 mice per group.

The percentage of power contained within the delta (1-4Hz) and theta (4–8.5Hz) frequency ranges of EEG are important components associated with the maturing brain and sleep patterns. Overall, the percentage of delta power decreased and the percentage of theta power increased during development independent of vigilance state (Fig 5). During wakefulness, the percentage of delta power decreased until P17, whereas the percentage of theta power increased starting at P14 (Fig 5A). The relative percentage of delta power associated with NREM sleep bouts decreased until P17 while the percentage of theta power is increased at P14 (Fig 5B). REM sleep featured more limited decreases in relative delta power after P10 and increases in theta after P12.

## Discussion

In this study, we performed a longitudinal assessment of EEG and vigilance states in the developing neonatal mouse. Previous studies have examined different aspects of EEG and sleep ontogeny in immature rodents, particularly in rats [8–14], but few EEG studies of vigilance states exist in neonatal mice [15–17] and to our knowledge, none have utilized a longitudinal approach in developing mice. As mice are commonly used for genetic studies of human development and disease, we attempted to provide a relatively comprehensive examination of EEG features and vigilance state across development of the neonatal mouse. The main findings are: 1) Prior to P10, the EEG background is primarily discontinuous, with no obvious state change, 2) By P10, EEG specific vigilance states became distinguishable, 3) The EEG becomes progressively more continuous between P10 and P14, 4) The percentage of time in the awake state increases and time in REM sleep correspondingly decreases from P10 to P21, 5) The number of transitions between states (except NREM to awake) decreases and the individual bout durations correspondingly increase from P10 to P21, and 6) The total power increases throughout development, while the relative delta power decreases and theta power increases, in all states throughout development. Overall, our findings in mice are consistent with developmental changes in comparable studies of other rodents, particularly rats, as well as in human, as discussed below, but possibly constitute the most comprehensive longitudinal EEG study in the neonatal mouse, including detailed vigilance state evaluation and spectral analysis followed serially in the same set of mice.

In infants and young mammals the brain undergoes rapid development and cortical EEG can provide a means to assess some of these developmental changes. In preterm human infants and young neonatal rodent models, cortical EEG features low voltage discontinuous activity that progressively develops into a continuous pattern in a state-dependent fashion [2, 7, 9, 22]. In preterm human infants, cortical EEG features a gradual increase in continuity first in the awake state, allowing for initial state differentiation around 32 weeks of gestational age, with near continuity in all vigilance states emerging by 40 weeks gestational age [3, 5, 6, 23]. Neonatal rodent models have shown similar EEG patterns with a general increase in continuity from P4- P12 [9, 15, 17, 22] and EEG differentiation of vigilance state activity around P10-11 in rat models [8, 12, 14, 24, 25]. Our EEG assessment of neonatal mice provides a similar developmental trend of an increase in continuity from P9 to P10 and initial vigilance state differentiation at P10, with continuous EEG emerging during awake and REM at P10 and near continuous EEG in NREM around P12.

Sleep is the dominant activity in human infants and young mammals with increased sleep/wake cycle duration and increased wakefulness occurring with maturity [4]. In neonatal rodents, wakefulness initially consists of short transitioning periods between sleep episodes marked by brief arousals or short bouts of wakefulness. Longer bouts of wakefulness occur but are limited in young neonatal rodents [26, 27]. Developing neonatal and preweaning rodents (P8-P21) display a progressive increase in wake time and wake bout duration [10, 16, 17, 26]. Conversely, during neonatal development NREM and REM sleep periods are shorter and less consolidated with significantly higher proportion of REM sleep than later in life. After EEG slow wave activity emerges until around weaning a precipitous decline in percentage of time spent in REM sleep occurs in rats as wakefulness increases [10, 13, 28]. Individual REM bouts decrease and NREM sleep bouts increase as sleep becomes more consolidated over course of neonatal development and into young adulthood. After EEG differentiation and depending on methods used to assess vigilance state, the percentage of time in NREM sleep increases but at a much slower pace and continues to increase after circadian and diurnal rhythms develop until around the 4^th^ week of life [11, 29, 30].

In our study, mice follow a similar progression over the neonatal and infantile periods with increased wakefulness and awake bout durations and corresponding decreased REM sleep. After P14, REM sleep periods follow longer NREM bouts as sleep becomes more consolidated and adult-like. In contrast with most previous rat studies, however, we found a higher percentage of NREM sleep relative to REM sleep in the neonatal and younger infantile mice. Consistent with Seelke and Blumberg [12], using a combination of EEG and EMG to assess vigilance state we found that percentage of recorded time in NREM sleep may be underestimated in previous studies because short NREM bouts occur during active sleep periods within the short sleep cycles of P10-P12 mice. Vogel et al. [13] also states that some periods of behavioral active sleep contain high percentage of delta activity in cortical EEG which we would have scored as NREM. We found infantile mice displayed a gradual (not significant) increase in percentage of NREM sleep and wakefulness replaced much of REM sleep over the developing period. Finally, the study by Daszuta and Gambarelli [17] most likely represents the most complete previous developmental investigation of vigilance states in mice and also finds a larger percentage of NREM sleep than REM, but did not include a longitudinal design or spectral analysis. Our study extends these findings by performing a more detailed longitudinal analysis of awake-sleep transitions and spectral power analysis in mice.

In addition to the initial increase in continuity, cortical EEG in the infantile rat increases in total power across all frequencies during the P9 to P21 period independent of vigilance state [8, 10, 12, 14, 31]. State specific EEG patterns also show considerable increases in power over this time period. Rat cortical EEG during NREM sleep displays at least a doubling of power particularly in the delta range from P11-P14 period and a more gradual increase in power over the remainder of the infantile period. Around the second postnatal week, rat cortical EEG displays more theta activity and higher frequencies as the brain develops. Particularly, during REM sleep periods the emergence of more uniform theta oscillations develop around 5-6Hz and further shift of the theta patterns to higher portions of the theta band as more adult-like EEG develops. While there have been minimal previous data on state-dependent power changes in mice, our study confirms similar trends of an overall power increase, but relative decreased percentage of delta and increased percentage of theta power, in the developing mouse.

Overall, our study in mice closely supports most of the developmental EEG and sleep ontogeny patterns previously characterized in neonatal rats. Limitations of our study include recording sessions restricted to a few hours due to neonatal pup size, mouse dam dependence, and technical limitations associated with tethered EEG recordings in pre-weanling mice. We also only recorded during the light phase, which could introduce bias secondary to possible diurnal effects or circadian rhythms. However, prior studies indicate that rats generally do not demonstrate diurnal variation or consolidated circadian rhythms until P17 [11, 14, 32]. Our technique for acquiring EEG and EMG in the developing mouse allowed for repeated, longitudinal EEG and sleep assessments in mice during this critical period of brain development. In addition, although we found no obvious sex differences in EEG or sleep ontogeny in this initial study, the study was not designed or powered to detect sex differences as this should be a subject of future studies. Furthermore, while our mice were on a mixed genetic background, genetic strain could be a potential modulator of sleep and EEG development and also deserving of more detailed comparative studies in the future [17]. As this was purely a descriptive study, mechanisms underlying sleep ontogeny and circadian development can be addressed following similar longitudinal recording protocols. Finally, this study provides important control data in investigations of the effects of neurological disease on EEG and sleep characteristics on the neonatal brain, such as neonatal hypoxia-ischemia, seizures, or numerous genetic disorders [15, 33–40].

## Supporting information

S1 Dataset Compiled raw data used for analysis of developmental changes in EEG patterns of the neonatal mouse. (XLSX)
